# The influence of gratitude and personality traits on career resilience and career success among college students

**DOI:** 10.3389/fpsyg.2024.1340200

**Published:** 2024-04-24

**Authors:** Kai Zhao, Wenna Ji

**Affiliations:** ^1^Mokwon University, Daejeon, Republic of Korea; ^2^Enrollment and Employment Center, Nanning College of Technology, Guilin, Guangxi, China

**Keywords:** gratitude, personality traits, extraversion, conscientiousness, career resilience, career success

## Abstract

**Introduction:**

The study examines the interplay between gratitude and career success, with career resilience as the mediating mechanism and personality traits, i.e., conscientiousness and extraversion, as moderating factors. The overarching goal is to enhance our understanding of the complex dynamics that influence career outcomes of college students in China.

**Methods:**

Data are gathered through a survey-based technique, capturing responses from a diverse sample of participants. The analysis employs Structural Equation Modeling (SEM) to explore the relationships among gratitude, career resilience, personality traits, and career success.

**Results:**

The results reveal that gratitude impacts students’ career success through the mediating mechanism of career resilience. In addition, conscientiousness and extraversion are found to positively intervene the direct effect between gratitude and career resilience and the indirect effect between gratitude and career success through career resilience.

**Discussion:**

The findings offer valuable insights for individuals, organizations, and career development practitioners, emphasizing the importance of cultivating gratitude and recognizing the differential impact of personality traits on this process. As organizations seek to foster resilient and successful career paths, acknowledging these nuanced dynamics can inform targeted interventions and strategies.

## Introduction

In the pursuit of academic and professional achievements, career success emerges as a crucial goal for college students, significantly influencing their future prospects. This objective is particularly paramount among Chinese college students, where the pursuit of career success gains heightened importance due to the intensely competitive and dynamically evolving job market in China (Yang et al., 2022; Zhong et al., 2022). Prior research has delved into various antecedents of career success, highlighting the roles of academic achievements (Kraimer et al., 2019), networking (Davis et al., 2020), and personality traits such as conscientiousness and extraversion (Spurk et al., 2019; Lei et al., 2021) in shaping individuals’ career trajectories. Despite these insightful contributions, a significant gap remains in understanding the influence of gratitude on career outcomes. Gratitude, a profound appreciation for the positive aspects of life, is well-documented for its beneficial impact on overall well-being (Bono et al., 2020). However, its direct implications for enhancing career success, especially among the youth in China, have not been extensively explored.

To address this research gap, the present study is designed to illuminate the nuanced relationship between gratitude and career success among Chinese college students. Career success is conceptualized as the progress toward and achievement of educational and professional development objectives (Pshembayeva et al., 2022). This includes the acquisition of knowledge and skills relevant to future employment, the development of professional identity and purpose, and the enhancement of employability attributes, such as career adaptability and readiness for the transition to the workforce. This construct is particularly vital for college students as it signifies the culmination of their academic efforts and marks a critical juncture in their professional lives. The emphasis on gratitude arises from its recognized capacity to engender a positive perspective and psychological fortitude (Bono et al., 2022), which are crucial for successfully navigating the complexities of the contemporary job market. This investigation proposes that gratitude can significantly influence the career trajectories of students by fostering an optimistic outlook and enhancing their adaptability and perseverance in the face of career challenges. By weaving together gratitude and career success, the current research endeavors to enrich the career development discourse with fresh insights, elucidating the role of positive psychological attributes in advancing career outcomes.

In addition, the study examines the intermediary role of career resilience in the direct relationship between gratitude and career success. The rationale for investigating the mediating mechanism of career resilience in the context of Chinese college students stems from the critical importance of understanding how individuals navigate the complexities and challenges inherent in their career trajectories (Rivera et al., 2021; Tokbaeva and Achtenhagen, 2023). Career resilience, defined as the ability to adapt positively to adverse career events, setbacks, and uncertainties (Brewer et al., 2019), emerges as a key factor that can significantly influence one’s capacity to rebound from setbacks and sustain a successful career path (Su et al., 2022). The study of career resilience is paramount, especially in the context of Chinese students, given the highly competitive and rapidly changing nature of the job market (Zeng et al., 2022). To delve into the intricacies of career resilience, the transactional theory of stress and coping (TTSC) provides a theoretical foundation. The TTSC theory proposed by Lazarus and Folkman (1984) posits that individuals engage in ongoing transactions with their environment, appraising and reappraising stressors while employing coping mechanisms to manage the challenges they encounter. Understanding career resilience through the lens of this theoretical framework allows for a nuanced exploration of how Chinese college students navigate the stressors inherent in their academic and professional pursuits, shedding light on the adaptive processes that contribute to sustained career success. Investigating the mediating role of career resilience in the relationship between gratitude and career success provides a holistic perspective on the dynamic interplay of these factors in the unique context of Chinese higher education and career development.

Moreover, the study anticipates that personality traits: conscientiousness and extraversion serve as the moderating factors that underpin the association between gratitude and career resilience (and then career success). The intervening role of personality traits recognizes the individual differences that shape how college students respond to the interplay between gratitude, career resilience, and career success. Personality traits, specifically conscientiousness and extraversion, are crucial factors influencing how individuals approach their academic and professional endeavors (Zandi Payam and Mirzaeidoostan, 2019). The selection of these particular personality traits is grounded in their established psychological significance and their proven impact on behavior and outcomes in both academic and professional settings. Conscientiousness, characterized by diligence, reliability, and a strong sense of duty, directly influences an individual’s ability to set, pursue, and achieve career-related goals with persistence and discipline (Diener and Lucas, 2019). Extraversion, marked by sociability, assertiveness, and enthusiasm, facilitates networking and interpersonal interactions, which are critical for navigating and succeeding in competitive job markets (Oshio et al., 2018).

The integration of conscientiousness and extraversion as moderating factors is rooted in the premise that these traits significantly affect how individuals engage with their environment, process experiences, and implement coping strategies (Steiner et al., 2023). For instance, conscientious students may be more adept at leveraging gratitude to enhance their career resilience, applying a methodical approach to overcoming obstacles and capitalizing on opportunities. Similarly, extraverted students might use their gratitude-driven positive outlook to foster relationships and networks that bolster their career progress.

This nuanced exploration is informed by the theoretical and empirical underpinnings that suggest personality traits play a pivotal role in shaping individuals’ interactions with their psychological resources and external opportunities. By examining how conscientiousness and extraversion moderate the relationship between gratitude and career outcomes, the study aims to provide a deeper, more detailed understanding of the psychological mechanisms at play. This approach does not merely recognize individual differences as a factor in career development but actively investigates how these differences mediate the effect of gratitude on career resilience and success (Jiang et al., 2019). Therefore, the inclusion of personality traits as moderators seeks to elucidate the interplay of internal dispositions and external influences, offering valuable insights into personalized strategies for career development within the context of Chinese higher education. This alignment with contemporary research reinforces the significance of a multifaceted exploration of career outcomes, where personality traits are considered integral to the formulation of effective, individualized career development interventions.

The ensuing sections of this paper systematically explore the theoretical underpinnings and empirical investigations that frame our study. Initially, we delve into a comprehensive literature review, focusing on the transaction theory of stress and coping, to contextualize the mediating role of career resilience and the influence of gratitude and personality traits on career success. This is followed by a detailed exposition of our methodology, outlining the sampling procedures, research instruments, and analytical techniques employed. Subsequent sections present the results of our analysis, revealing the relationships among the study variables. Finally, the discussion synthesizes our findings within the broader spectrum of career development research, highlighting theoretical implications, practical applications, and avenues for future inquiry in the context of Chinese higher education.

## Literature review

### Transaction theory of stress and coping

The TTSC, proposed by Lazarus and Folkman (1984), is a prominent psychological framework that offers valuable insights into the dynamic processes through which individuals perceive and respond to stressors. According to TTSC, stress is a result of an individual’s subjective appraisal of an event or circumstance and the coping strategies they employ to manage it (Meraz et al., 2023). This theory distinguishes between primary appraisal, involving the evaluation of a situation’s significance, and secondary appraisal, focusing on one’s perceived ability to cope (Lee and Roberts, 2018). Coping mechanisms can be problem-focused, aimed at directly addressing the stressor, or emotion-focused, designed to manage the emotional response. Previous studies applying TTSC have highlighted its efficacy in understanding how individuals navigate stressors in various life domains, including academia and the workplace (Shen and Slater, 2021).

In the context of career development, TTSC has been instrumental in elucidating the processes by which individuals adapt to and cope with challenges, shaping their overall career outcomes. For instance, research has shown that individuals with effective coping strategies, as guided by TTSC, are better equipped to manage career-related stressors, leading to increased job satisfaction and success (Lazarus and Folkman, 1984). In our study, TTSC serves as a conceptual underpinning to explore how Chinese college students appraise and cope with career-related stressors, particularly through the lens of gratitude, personality traits, and career resilience. Incorporating gratitude into the TTSC framework, our study extends its application to the context of career development among Chinese college students. Gratitude, a positive emotional state acknowledging the benefits received from others (Kaniuka et al., 2021), can significantly influence both the primary and secondary appraisal processes identified by TTSC. In the realm of career development, gratitude may prompt individuals to perceive career-related challenges more positively, potentially viewing them as opportunities for growth rather than insurmountable obstacles. This optimistic appraisal could enhance the effectiveness of coping strategies, as individuals grounded in gratitude are likely to engage in more constructive problem-solving and emotional regulation practices.

Therefore, in our exploration of how gratitude, alongside personality traits such as conscientiousness and extraversion, affects career resilience and success, TTSC provides a robust theoretical lens through which the mediating effects of career resilience can be understood. By weaving gratitude into the fabric of TTSC, we aim to illuminate the nuanced ways in which a grateful disposition can fortify students’ resilience to career stressors, thereby facilitating a pathway to career success. This integration seeks to offer a holistic view of the psychological assets that buffer against career-related stress and contribute to the thriving career trajectories of Chinese college students.

### Gratitude and career success

The relationship between gratitude and career success accentuates a transformative perspective in the pursuit of career development. Gratitude, characterized by a profound appreciation for received benefits and positive life experiences, extends beyond mere emotional well-being to significantly impact an individual’s professional trajectory (Armenta et al., 2017). This positive psychological construct fosters resilience, adaptability, and a proactive orientation toward career challenges and opportunities. In the context of career success, gratitude equips individuals with the mental and emotional fortitude to navigate the complexities of their professional paths (Arora and Rangnekar, 2016), which enables them to seize opportunities, cultivate meaningful relationships, and approach their career aspirations with optimism and persistence. Consequently, individuals who practice gratitude are likely to experience enhanced job satisfaction, greater professional achievements, and a more fulfilling career journey (Kong et al., 2015; Saylors et al., 2020). By promoting a positive outlook and a gratitude-centric mindset, individuals can transform their career experiences, aligning personal values with professional goals, thereby achieving a heightened sense of career success. This relationship between gratitude and career success highlights the pivotal role of positive psychological resources in shaping not just the trajectory but the quality of one’s professional life. The current study seeks to explore the boundary effects of the gratitude–career success relationship by analyzing the mediating role of career resilience and moderating role of personality traits.

## Hypotheses

### Linking gratitude with career resilience

The link between gratitude and career resilience is a crucial aspect of our study, as it delves into the dynamics that shape the adaptive responses of Chinese college students to career-related challenges. Gratitude, characterized by a positive recognition and appreciation of life’s positive aspects, serves as a psychological resource that can potentially enhance individuals’ ability to cope with setbacks and uncertainties in their professional journeys (Kong et al., 2015). As students encounter obstacles or face uncertainties in their career paths, a disposition of gratitude may contribute to a more optimistic outlook, fostering a mindset conducive to resilience (Bono et al., 2022). When individuals experience gratitude, they may be more likely to view challenges as opportunities for growth and learning rather than insurmountable obstacles. This positive cognitive appraisal aligns with the core principles of career resilience, which involves the ability to adapt positively to adverse career events (Ismail and Rishani, 2018). Gratitude, by fostering a positive mindset, may thus play a pivotal role in influencing how Chinese college students navigate and bounce back from setbacks in their academic and professional pursuits. Previous studies have acknowledged the potential links between positive psychological constructs, including gratitude, and adaptive coping mechanisms. For instance, the work of Mary and Patra (2015) suggests that gratitude is associated with increased resilience in the face of stress. Extending this understanding to the career domain, our study aims to explore the specific pathways through which gratitude contributes to the cultivation of career resilience among Chinese college students.

Furthermore, building on the TTSC, which posits that stress results from individuals’ appraisals of their circumstances and their subsequent coping responses (Meraz et al., 2023), gratitude can be understood as a critical resource that influences these appraisals in a positive way. Gratitude encourages a positive reinterpretation of challenging situations, which can lead to more effective coping strategies (Bono et al., 2022). Specifically, gratitude may promote a problem-focused coping approach (Gaeta et al., 2021), where individuals are more likely to take proactive steps toward goal achievement and career advancement, viewing obstacles as manageable and as opportunities for growth. Thus, within the TTSC framework, gratitude acts as a lever, positively affecting both the appraisal of career challenges and the selection of coping mechanisms, ultimately paving the way for career success. Thus, we hypothesize that:

*H1*. There is a significant positive relationship between gratitude and career resilience.

### Linking career resilience with career success

The connection between career resilience and career success forms a critical nexus in understanding how individuals, particularly Chinese college students, navigate the complexities of their professional journeys. Career resilience, defined as the ability to adapt positively to adverse career events, provides individuals with the psychological resources necessary to persevere and rebound from setbacks (Finley, 2018). In the context of career success, the adaptive capacities inherent in career resilience become instrumental. Individuals who can effectively cope with challenges, setbacks, and uncertainties in their careers are likely to exhibit greater perseverance, adaptability, and a proactive approach (Seibert et al., 2016), all of which are conducive to achieving sustained success. Numerous studies have underscored the positive impact of career resilience on various indicators of career success. For instance, research by Xia et al. (2011) found that career resilience positively correlates with career satisfaction and job performance. Additionally, individuals with higher levels of career resilience are more likely to demonstrate career progression, achieve career goals, and sustain overall job satisfaction (Han et al., 2021). In the context of Chinese college students, understanding the link between career resilience and success is crucial.

In addition to these arguments, we draw our insights on the basis of TTSC (Lazarus and Folkman, 1984). Career resilience, as conceptualized within the TTSC framework, represents an individual’s adaptive capacity to navigate career-related stressors effectively (Longoria, 2023). This capacity is cultivated through a combination of problem-focused and emotion-focused coping strategies (Zhang et al., 2022), enabling individuals to maintain or regain their footing in the face of challenges. The theory suggests that the way individuals appraise career stressors—viewing them as manageable and as opportunities for growth (Lazarus and Folkman, 1984)—can significantly enhance their resilience. This resilience, in turn, facilitates career success by empowering individuals to pursue their career goals persistently, adapt to changes, and recover from setbacks more swiftly and effectively.

The linkage between career resilience and career success, therefore, is underpinned by the transactional processes outlined in TTSC. Individuals with high career resilience are likely to engage in more effective coping strategies, as they are better prepared to assess stressors positively and employ adaptive responses. This proactive engagement with career challenges not only mitigates the adverse effects of stress but also promotes career advancement, satisfaction, and achievement. Thus, we hypothesize that:

*H2*. There is a significant positive relationship between career resilience and career success.

### The mediating role of career resilience

Subsequently, the study predicts the mediating role of career resilience between gratitude and career success. Within the TTSC framework, career resilience assumes a pivotal mediating role in our study, providing insights into the dynamic processes through which gratitude influences career success among Chinese college students. As per TTSC, individuals engage in continuous transactions with their environment, appraising stressors and employing coping strategies to manage challenges (Lazarus and Folkman, 1984). In this context, gratitude, as a positive cognitive and emotional response, is likely to influence the primary appraisal of career-related stressors, potentially framing them as opportunities for growth.

Career resilience, acting as a mediating factor in this transactional process, reflects the adaptive coping mechanisms individuals deploy to navigate these stressors effectively. Gratitude, by fostering a positive outlook and adaptive mindset, is expected to influence how Chinese college students appraise and cope with challenges in their academic and professional pursuits. This positive cognitive appraisal, in turn, is likely to bolster career resilience, enabling individuals to bounce back from setbacks and persist in the face of adversity. By exploring the mediating role of career resilience within the TTSC framework, our study seeks to unravel the nuanced pathways through which gratitude, guided by cognitive appraisals and coping mechanisms, contributes to sustained career success among Chinese college students. This approach aligns with TTSC’s emphasis on the transactional nature of stress and coping, providing a comprehensive understanding of the interplay between psychological factors and adaptive responses in the pursuit of successful careers. Thus, we hypothesize that:

*H3*. Career resilience mediates the relationship between gratitude and career success.

### Moderating role of personality traits

Personality traits, as enduring dimensions of individual differences in behavior, cognition, and emotion, are fundamental to understanding how individuals approach and navigate life’s challenges (Diener and Lucas, 2019). Within the Five-Factor Model (FFM) of personality, conscientiousness and extraversion are two prominent traits. Conscientious individuals are characterized by traits such as organization, reliability, and goal-directed behavior, reflecting a proactive and disciplined approach to tasks (Oshio et al., 2018). Extraversion encompasses sociability, assertiveness, and a propensity for positive engagement in social activities, signifying a more outgoing and energetic disposition (Arora and Rangnekar, 2016). Previous research has established the crucial role of conscientiousness and extraversion in shaping adaptive responses to stressors and challenges. For instance, conscientiousness has been linked to effective coping strategies and resilience in various life domains (Meléndez et al., 2020). Likewise, extraversion, with its emphasis on social engagement and assertiveness, is associated with enhanced interpersonal skills and positive outcomes in social contexts (Lo et al., 2022).

In the context of our study, conscientiousness and extraversion are proposed as moderators in the relationship between gratitude and career resilience among Chinese college students. Drawing on existing literature, research by Baek (2017) suggests that conscientious individuals may exhibit a greater capacity to cope with stressors, emphasizing the importance of conscientiousness in shaping adaptive responses. Additionally, the study of Shaheen et al. (2015) demonstrates that extraversion is linked to positive outcomes in the workplace, indicating its potential to influence adaptive behaviors and resilience. In the context of career development, research by Sari (2020) highlights the role of conscientiousness in predicting job performance, suggesting its relevance to outcomes in professional domains. Besides, extraversion has been associated with success in leadership roles and positive workplace outcomes (Wilmot et al., 2019). Applying these insights to our study, we posit that conscientiousness and extraversion might moderate the relationship between gratitude and career resilience among Chinese college students. Conscientious individuals, with their disciplined and goal-oriented nature, may amplify the positive effects of gratitude on career resilience, aligning with their proactive approach to challenges. Similarly, individuals high in extraversion, with their social acumen, may leverage interpersonal resources to enhance the positive impact of gratitude on building resilient networks. By exploring these moderating effects, our study contributes to the growing body of literature on the role of personality traits in shaping adaptive responses to stressors and challenges, particularly within the unique context of Chinese higher education and career development.

Integrating the TTSC (Lazarus and Folkman, 1984) with the concept of personality traits as moderators offers a comprehensive framework to understand how individual differences influence the stress-coping process and, subsequently, career outcomes. TTSC posits that stress and coping involve an individual’s appraisal of stressors and their capacity to manage these challenges through various coping strategies (Saylors et al., 2020). Personality traits can significantly affect both components of this process: the appraisal of stressors and the selection of coping mechanisms.

The moderating role of personality traits, such as conscientiousness and extraversion, becomes particularly relevant in this context. Individuals high in conscientiousness, characterized by diligence, responsibility, and organized behavior, may appraise potential career stressors more positively, seeing them as opportunities for growth and development (Sari, 2020). This positive appraisal could lead to more effective problem-focused coping strategies, aligning with TTSC’s emphasis on the adaptive management of stress. Conscientious individuals are likely to be proactive in planning and executing actions to mitigate stressors, thereby enhancing their career resilience and success.

Similarly, extraversion, which encompasses sociability, assertiveness, and enthusiasm, may influence the emotional-focused coping component of TTSC. Extraverted individuals tend to have larger social networks and are more likely to seek support from others when faced with stress (Steiner et al., 2023), an emotion-focused coping strategy. This propensity can serve as a buffer against the negative impacts of career-related stress, facilitating better psychological well-being and, by extension, more positive career outcomes. Thus, we hypothesize that:

*H4*. Personality traits: (a) conscientiousness and (b) extraversion, moderate the relationship between gratitude and career resilience, such that individuals high in these traits will more likely be exhibiting superior career resilience.

Integrating the moderating role of personality traits in Hypothesis *H4* with the mediation model (Hypothesis *H3*), we propose the following hypothesis:

*H5*. Personality traits: (a) conscientiousness and (b) extraversion, moderate the relationship between gratitude and career success through career resilience, such that individuals high in these traits will more likely be experiencing superior career success via career resilience.

**Figure 1:**
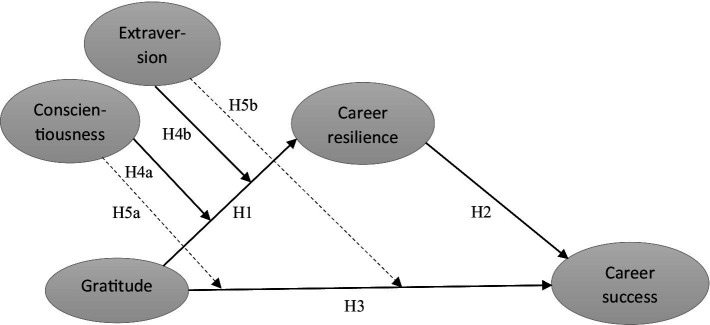
Conceptual model.

The proposed conceptual model is shown in Figure 1.

## Methodology

### Sampling and procedures

In this study, data were collected from students enrolled in Chinese educational institutions. This adheres to the strategic decision driven by the relevance of the proposed model to the cultural and educational context of China. Ample research suggests that cultural nuances significantly influence individuals’ perceptions of gratitude, coping mechanisms, and personality traits (Brewer et al., 2019). By focusing on Chinese college students, our study aligns with the growing recognition that cultural backgrounds can shape the dynamics of psychological processes and responses (Bono et al., 2022). This choice ensures that the findings resonate with the specific challenges and opportunities inherent in the Chinese higher education system, contributing to a more contextually grounded understanding of the relationships within the proposed model.

To ensure the representativeness of the sample, random sampling was employed. According to Saunders (2012), random sampling possesses the capacity to yield a sample that mirrors the characteristics of the larger population. This approach enhances the generalizability of the study’s findings to the broader population of Chinese college students. Besides, by employing random sampling, the study aims to minimize selection bias and strengthen the external validity of the results.

Moreover, the data collection process utilized a cross-sectional research design. The cross-sectional design allows for the simultaneous examination of gratitude, personality traits, career resilience, and success within a specific timeframe. The use of cross-sectional data aligns with the study’s goal of providing a comprehensive but time-specific understanding of the proposed model. In total, 500 survey responses were distributed, with 444 returned and processed for data analysis, ensuring a robust dataset for examining the interplay of these variables among Chinese college students.

The sample exhibited a balanced gender distribution, with 53% identifying as male and 47% as female. In terms of academic standing, 37% were undergraduate students, 40% pursuing graduate studies, and 23% engaged in post-graduate programs. The participants’ average age was 26 years, with a standard deviation of 5.23. Regarding the field of study, the respondents represented a broad spectrum of disciplines, with 32% affiliated with social sciences, 39% with natural sciences, and 29% with engineering.

### Research design

The research design of our study is structured as a quantitative investigation employing a cross-sectional survey methodology. This approach was selected for its efficiency in capturing a snapshot of the relationships between gratitude, personality traits, career resilience, and career success among Chinese college students at a specific point in time. By utilizing structured questionnaires, the study aims to collect data from a large, diverse sample, enabling the statistical analysis of variables and the exploration of potential correlations and influences. The choice of a cross-sectional design facilitates the examination of the proposed hypotheses within a defined timeframe that offers insights into the dynamics at play without the need for longitudinal tracking. This methodology aligns with the study’s objectives to explore complex psychological constructs and their impact on career development outcomes, and provides a robust framework for analysis and interpretation within the context of Chinese higher education.

### Research instrument

The research instrument to measure the hypothesized relationships among variables has been adapted from previous studies and measured on a 5-point Likert scale. The value 1 indicates a complete disagreement, while the value 5 indicates a complete agreement. Gratitude was measured using a 6-items scale developed by McCullough et al. (2001), with sample items: “I have so much in life to be thankful for” and “when I look at the world, I do not see much to be grateful for.” For measuring career resilience, Carson and Bedeian’s (1994) career commitment scale was used which consisted of 4-items. For instance, “the cost associated with my line of work/career field sometimes seem to great” and “given the problems I encounter in this line of work/career field, I sometimes wonder if the personal burden is worth it.” The mini-international personality item pool scale (Mini-IPIP) adapted from Donnellan et al. (2006) was used to measure personality traits: conscientiousness (4-items) and extraversion (4-items). For example, “get chores done right away” and “talk to a lot of different peoples.” Career success was measured with a 5-items scale developed by Greenhaus et al. (1990), with sample items: “I am satisfied with the success I have achieved in my career” and “I am satisfied with the progress I have made toward meeting my goals for the development of new skills.”

## Results

### Measurement model assessment

The study utilized the SmartPLS-SEM technique, a multivariate data analysis approach, to investigate the associations between the study’s variables (Ringle et al., 2022). In order to analyze the measurement model, the researchers performed an analysis to assess the psychometric properties: average variance extracted (AVE) to assess the convergent validity and Fornell-Larcker criterion and heterotrait-monotrait (HTMT) ratio to investigate the discriminant validity. To examine the presence of multicollinearity and mitigate any common method biasness (CMB), the study employed the variance inflation factor (VIF). The investigation yielded values below 3.3, indicating the absence of any problems related to CMB (Table 1).

**Table 1:** Collinearity assessment.

	Gratitude	Career resilience	Conscientiousness	Extraversion	Career success
Gratitude		1.95			1.10
Career resilience					2.01
Conscientiousness		2.42			2.15
Extraversion		0.87			1.03
Career success					

During the initial phase, an evaluation is conducted to assess the psychometric properties of the data, with the aim of establishing the study’s reliability and validity. According to Hair et al. (2018), the utilization of Cronbach’s alpha and composite reliability (CR) is recommended as appropriate approaches for evaluating reliability. Cronbach (1951) established that the appropriate range for the cutoff value falls within 0.70–0.95. Furthermore, it is recommended for social scientists to employ assessment methods to gage the robustness of their research, particularly by utilizing techniques like convergent and discriminant validity assessments. Convergent validity refers to the extent of a positive association between a measurement and additional measurements that evaluate the same underlying construct (Hair et al., 2018). The utilization of AVE scores is employed, where a value greater than 0.5 indicates a significant level of shared variance between the items or indicators and their corresponding concept. The analytical findings are displayed in Table 2, which shows that all the values are above the acceptable range.

**Table 2:** Reliability and convergent validity.

	Cronbach’s alpha	Composite reliability (rho_a)	Composite reliability (rho_c)	Average variance extracted (AVE)
Gratitude	0.735	0.788	0.838	0.566
Career resilience	0.796	0.839	0.872	0.650
Conscientiousness	0.812	0.859	0.886	0.613
Extraversion	0.865	0.920	0.923	0.601
Career success	0.803	0.848	0.865	0.631

Moreover, discriminant validity concerns the extent to which a specific construct can be distinguished from others using empirical measures (Hair et al., 2018). The Fornell-Larcker criteria (Table 3) indicates that all constructs—gratitude, career resilience, conscientiousness, extraversion, and career success—demonstrate adequate discriminant validity, as the square root of the Average Variance Extracted (AVE) for each construct (diagonal values) is greater than its correlation with any other construct (off-diagonal values).

**Table 3:** Fornell-Larcker criteria.

	Gratitude	Career resilience	Conscientiousness	Extraversion	Career success
Gratitude	0.752				
Career resilience	0.535	0.801			
Conscientiousness	0.381	0.583	0.782		
Extraversion	0.389	0.291	0.483	0.775	
Career success	0.692	0.601	0.774	0.699	0.794

In addition, to assess discriminant validity, researchers also rely on the HTMT ratio, as suggested by Henseler et al. (2009), which is considered a robust indicator of discriminant validity. When HTMT values are below 0.85 (HTMT < 0.85), it signifies that a variable possesses distinctive attributes and encapsulates a phenomenon that is not adequately represented by other variables within the model. The outcomes of this investigation are presented in Tables 3, 4.

**Table 4:** HMT ratio.

	Gratitude	Career resilience	Conscientiousness	Extraversion	Career success
Gratitude					
Career resilience	0.435				
Conscientiousness	0.353	0.573			
Extraversion	0.562	0.701	0.573		
Career success	0.635	0.691	0.694	0.489	

### Structural model assessment

After confirming the validity of the measurement model, the research progressed to assess the structural model in the subsequent phase. The study utilized a non-parametric bootstrapping method: bias-corrected and accelerated (BCa) technique. The BCa bootstrapping technique was implemented with a resample size of 5,000. The objective of this analysis was to get the values of path coefficients (β) and their accompanying *t*-values. The results of the present study, as illustrated in Table 5, indicate a statistically significant positive association between the gratitude and career resilience (β = 0.499; *t* > 1.95; *p* < 0.05), as well as between career resilience and career success (β = 0.541; *t* > 1.95; *p* < 0.05). These findings lend support to Hypotheses 1 and 2.

**Table 5:** Hypotheses testing.

	Path	β	*t*-value	*p*-value	*R* ^2^	Results
**Direct effects**						
H1	Gratitude career resilience	0.499***	9.540	0.000	0.447	Accept
H2	Career resilience career success	0.541***	10.262	0.000	0.551	Accept
**Mediation model (indirect effects)**						
H3	Gratitude career resilience career success	0.301***	4.494	0.001		Accept
**Moderated mediation model**						
H4a	Gratitude × conscientiousness career resilience	0.268***	3.117	0.003		Accept
H4b	Gratitude × extraversion career resilience	0.332***	5.732	0.000		Accept
H5a	Gratitude × conscientiousness career resilience career success	0.310***	6.800	0.000		Accept
H5b	Gratitude × extraversion career resilience career success	0.374***	7.512	0.000		Accept

***Significant at *p* < 0.05 (1.96).

Moreover, the study hypothesized that career resilience serves as a mediator in the relationship between gratitude and career success. The study obtained point estimates of the indirect effect using the BCa bootstrapping technique, using 5,000 resamples. The findings presented in Table 5 indicate that there is a substantial mediating impact of career resilience in the relationship between gratitude and career success. The statistical analysis shows that this relationship has a beta coefficient of 0.301, with a *t*-value greater than 1.95, and a *p*-value less than 0.05. These results support the existence of complementary mediation (Hair et al., 2018). Therefore, Hypothesis 3 has been confirmed.

**Figure 2:**
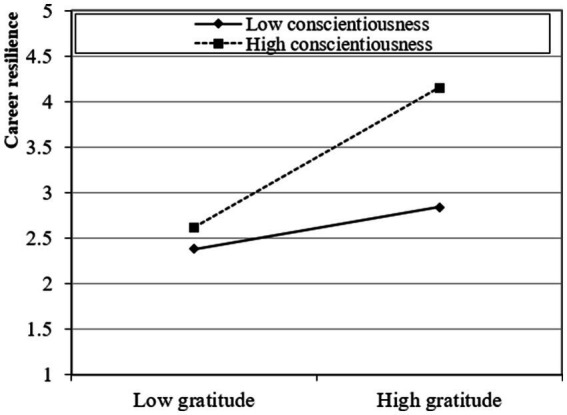
Moderation (1).

**Figure 3:**
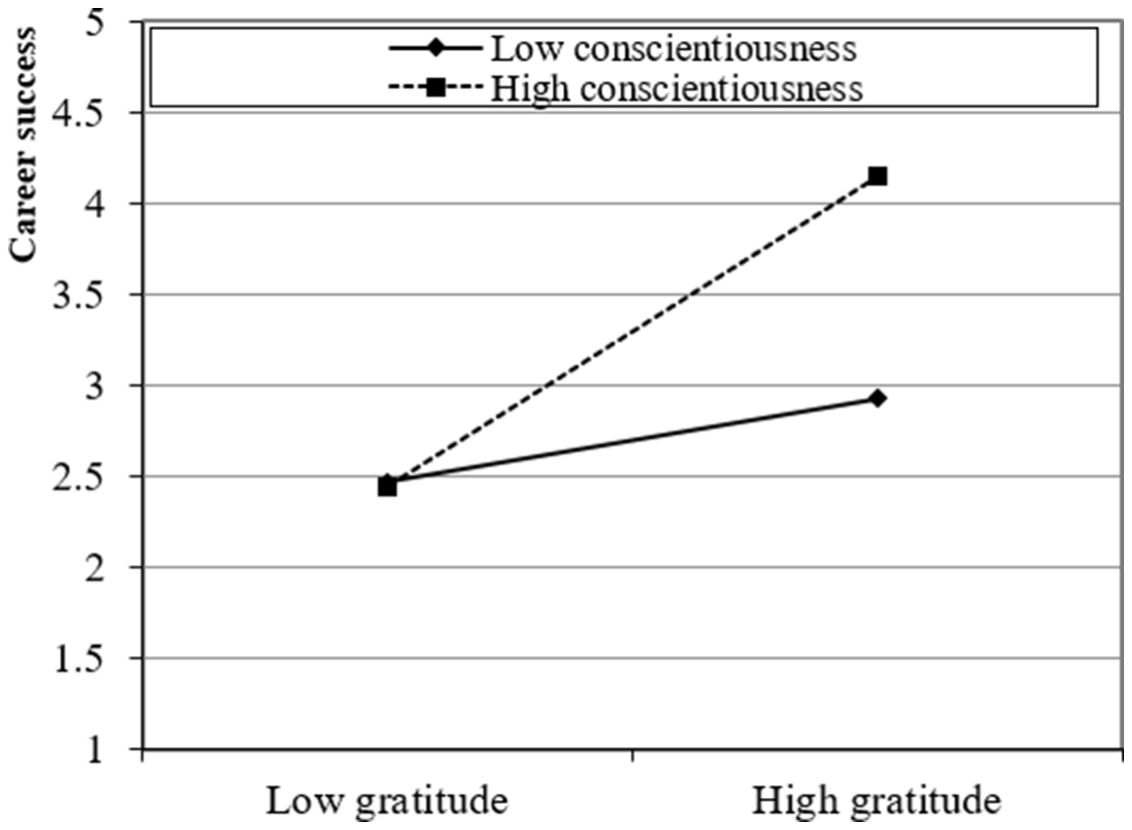
Moderated mediation (1).

**Figure 4:**
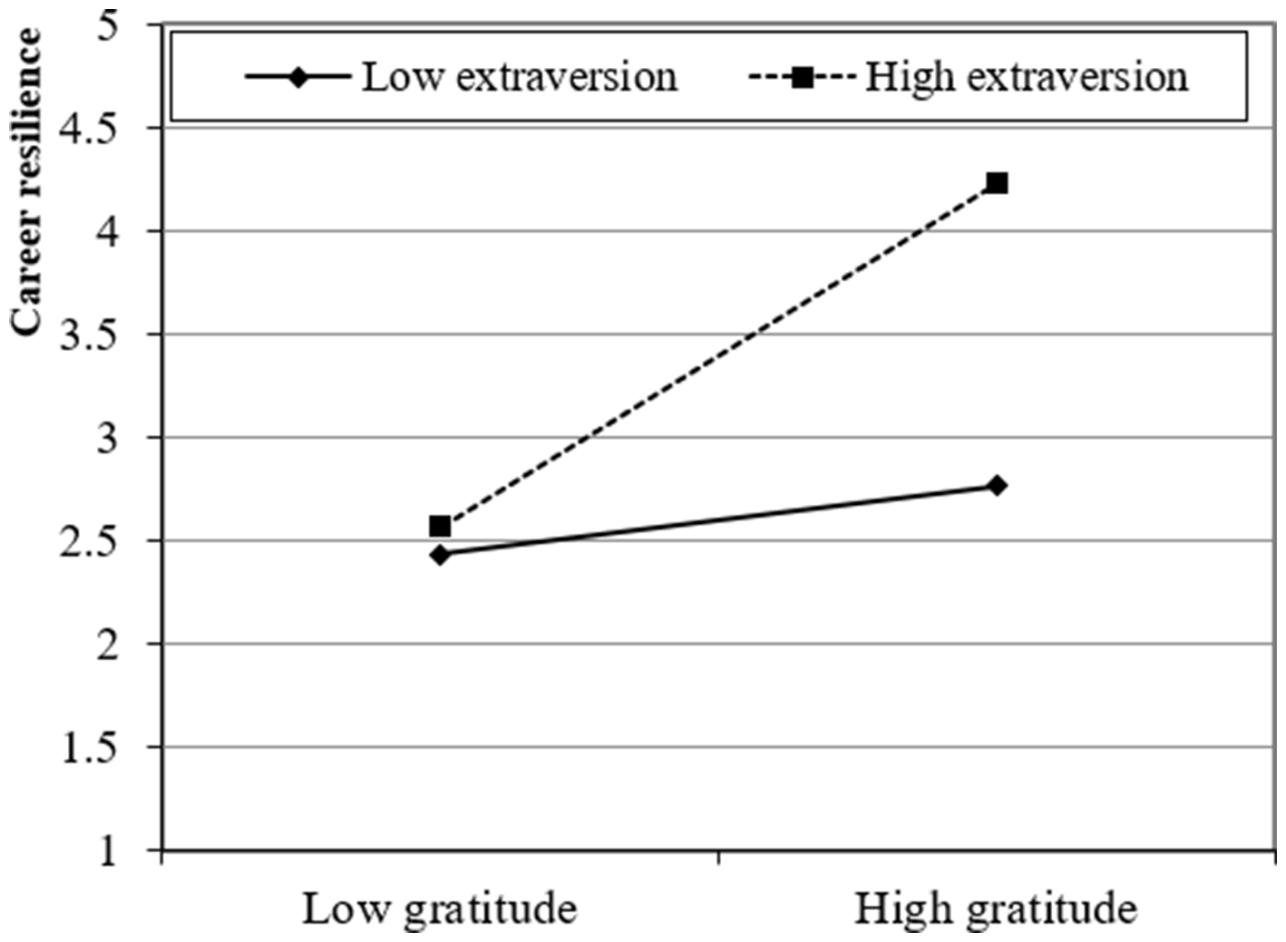
Moderation (2).

**Figure 5:**
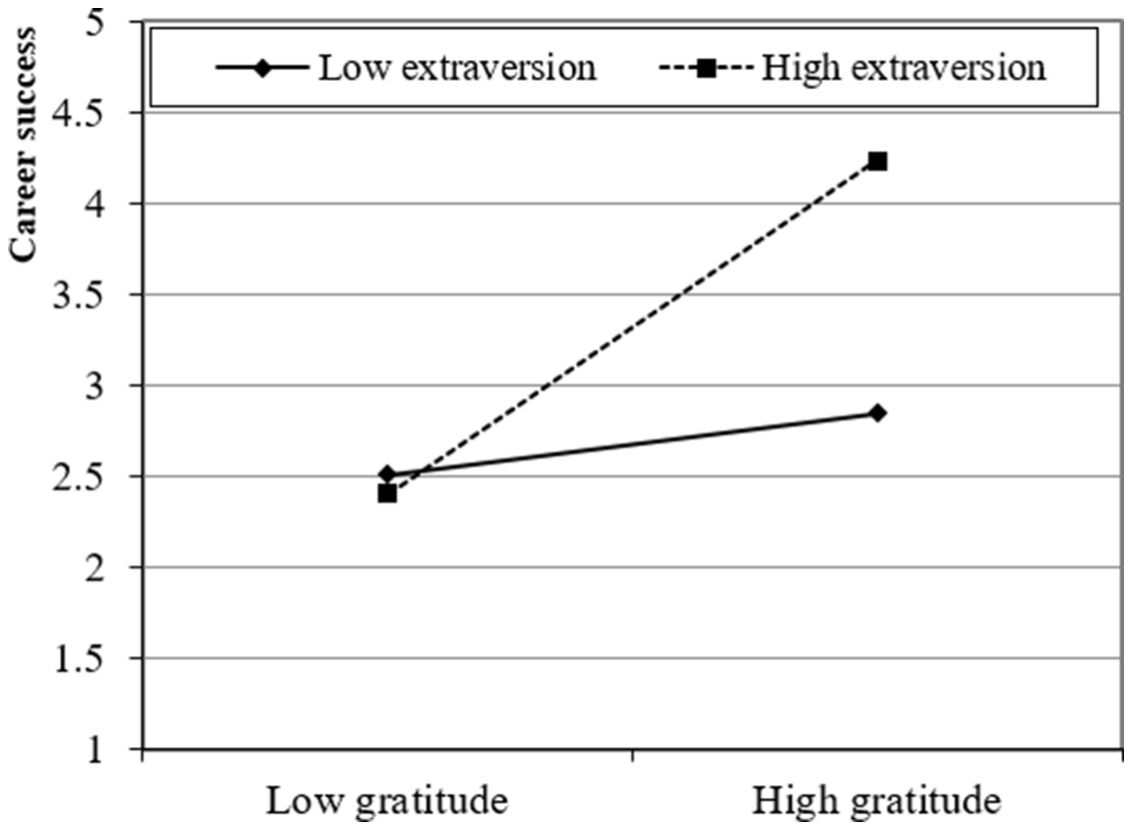
Moderated mediation (2).

The study presented a moderated mediation model and subsequently investigated the moderating influence of conscientiousness and extraversion using a two-stage approach (Hair et al., 2018). The findings of this analysis suggest that the interaction between gratitude and conscientiousness has a statistically significant positive effect on career resilience (β = 0.268; *t* > 1.95; *p* < 0.05) and career success through career resilience (β = 0.310; *t* > 1.95; *p* < 0.05), providing support for Hypotheses 4a and 5a. Moreover, the interaction between gratitude and extraversion has a statistically significant positive effect on career resilience (β = 0.332; *t* > 1.95; *p* < 0.05) and career success through career resilience (β = 0.374; *t* > 1.95; *p* < 0.05), providing support for Hypotheses 4b and 5b. Furthermore, following the recommendations proposed by Dawson (2014), the researcher also produced graphical depictions of fundamental slope interaction effects to enhance comprehension of the associations between gratitude and career success through career resilience. These associations are influenced by career resilience and are further moderated by conscientiousness and extraversion. Graphical representations in Figures 2–5 provide visual depictions of the effects of interaction terms. The utilization of simple slope analysis offers empirical evidence in favor of the hypothesized moderated mediation model. The findings of this study suggest that conscientiousness and extraversion play substantial roles in intensifying the relationship between gratitude and career success through career resilience, and vice versa.

Moreover, the researchers also examined the R^2^, a statistical measure that assesses the predictive capacity of the model. The computation involves determining the squared correlation between the observed and estimated values of a specific endogenous component (Hair et al., 2018). The observed values of R^2^ career resilience (0.447) and career success (0.551) indicate a moderate level of influence on the variability of endogenous variables. Furthermore, the researchers evaluated the predictive performance of the model by conducting tests on the root mean square error (RMSE). They then compared the RMSE values obtained from the partial least squares structural equation modeling (PLS-SEM) with those produced from the linear regression model (LM). The results indicate that the majority of the indicators of PLS-SEM RMSE demonstrate reduced prediction errors in comparison to LM RMSE, suggesting that the structural model possesses a reasonable level of predictive power. Finally, the study assessed the SRMR (Standardized Root Mean Square Residual) value to check the model fit in the study. An SRMR value of 0.062 falls below the commonly accepted threshold of 0.08, indicating a good fit between the model and the observed data. This suggests that the discrepancies between the predicted and observed covariance matrices are minimal.

## Discussion

The study investigates the interplay among gratitude, personality traits, career resilience, and career success among Chinese college students. The findings illuminate the mediating role of career resilience, illustrating how the positive emotions associated with gratitude contribute to the development of adaptive coping mechanisms crucial for navigating the challenges of academic and professional pursuits. Additionally, conscientiousness and extraversion emerge as significant moderators, influencing the strength and nature of the relationships within the proposed model. The study finds that conscientious individuals may intensify the positive effects of gratitude on career resilience, emphasizing the importance of proactive approaches to challenges. Similarly, individuals high in extraversion may leverage their social skills to enhance the positive impact of gratitude, creating a robust connection between gratitude, career resilience, and ultimately, career success. The discussion integrates these findings within the broader context of existing research on personality traits, gratitude, and career outcomes, offering insights for future interventions and strategies aimed at fostering resilient and successful career paths among Chinese college students. Our study’s findings are in harmony with previous studies and extends the existing body of knowledge in numerous ways, for instance:

*H1*: There is a significant positive relationship between gratitude and career resilience.

This hypothesis aligns with the broaden-and-build theory (Fredrickson, 2001), positing that positive emotions, such as gratitude, broaden individuals’ cognitive and behavioral repertoires, resulting in increased psychological resources. In the context of career development, the positive relationship between gratitude and career resilience suggests that the affective and cognitive benefits of gratitude may contribute to the cultivation of adaptive coping mechanisms crucial for navigating the challenges in one’s academic and professional journey. Besides, this finding resonates with the work of Armenta et al. (2017), which demonstrates the role of positive emotions, including gratitude, in enhancing resilience to stressors. By examining the influence of gratitude on Chinese students’ career resilience, our study advances the current line of inquiry in predicting students’ endurance to life’s challenges and their coping mechanism to deal with them based on their gratitude levels.

*H2*: There is a significant positive relationship between career resilience and career success.

The second hypothesis is in accord with the self-efficacy theory (Bandura, 1977), which proposes that individuals with a higher sense of resilience are more likely to possess a robust belief in their capability to overcome challenges and succeed in their careers. Moreover, the positive association between career resilience and career success aligns with research by Ahmad et al. (2019), Rossier et al. (2017), and Sharma and Tiwari (2023), emphasizing the positive impact of resilience on job performance. This theoretical perspective suggests that the adaptive responses fostered by resilience contribute to sustained success in the face of career-related obstacles. The combination of these hypotheses suggests the mediating role of career resilience in the association between gratitude and career success.

*H3*: Career resilience mediates the relationship between gratitude and career success.

The mediation hypothesis is consistent with the TTSC (Lazarus and Folkman, 1984), which highlights the ongoing interaction between individuals and their environment in shaping responses to stressors. The mediating role of career resilience suggests that the positive effects of gratitude on career success are channeled through individuals’ adaptive coping strategies. This is in line with previous studies emphasizing the mediating role of resilience in various life domains (Ang et al., 2022; Liang et al., 2023; Xiao et al., 2023), extending this understanding to the specific context of career development.

*H4*: Personality traits (a) conscientiousness and (b) extraversion, moderate the relationship between gratitude and career resilience, such that individuals high in these traits will more likely be exhibiting superior career resilience.

The moderating role of personality traits harmonizes with the resource-based model of stress and coping (Hobfoll, 1989), positing that conscientiousness and extraversion function as personal resources that enhance individuals’ coping capacity. The moderation effect implies that these traits amplify the positive impact of gratitude on career resilience. This resonates with Nandkeolyar et al.’s (2014), Pérez-Chacón et al. (2023), and Weiß et al. (2022) findings, indicating that conscientious individuals may deploy more effective coping strategies, thereby contributing to superior resilience. Subsequently, the study also finds that conscientiousness and extraversion positively intervene the association between gratitude and career success via career resilience, such that at high levels of conscientiousness and extraversion the indirect relationship between gratitude and career success via career resilience is more potent and vice versa. This is validated by our empirical findings such that:

*H5*: Personality traits (a) conscientiousness and (b) extraversion, moderate the relationship between gratitude and career success through career resilience, such that individuals high in these traits will more likely be experiencing superior career success via career resilience.

The fifth hypothesis extends the understanding of the TTSC (Lazarus and Folkman, 1984) by incorporating personality traits into the process. The moderation effect suggests that conscientiousness and extraversion influence not only the direct relationship between gratitude and career success but also the indirect pathway through career resilience. This echoes with previous research highlighting the influence of personality traits on positive workplace outcomes (Srivastava et al., 2015; Smith and DeNunzio, 2020; Mihalache and Mihalache, 2022) and reiterates the interplay of individual characteristics in shaping successful career paths.

## Conclusion

This study, rooted in the TTSC, illuminates the crucial roles of gratitude and personality traits in bolstering career resilience and success among Chinese college students. The findings reveal that gratitude acts as a significant psychological resource, enhancing individuals’ capacity to navigate career challenges effectively, while the moderating effects of personality traits such as conscientiousness and extraversion further underpin these outcomes. These insights advocate for the integration of positive psychological constructs into career development strategies, emphasizing the need for personalized approaches based on individual personality profiles.

### Theoretical implications

The theoretical implications of this study extend significantly within the realm of career development and positive psychology, and offers a nuanced understanding of how gratitude and personality traits influence career resilience and success. By integrating the TTSC with constructs of gratitude and personality traits, this research illuminates the interplay between an individual’s psychological resources and their career trajectory. Specifically, the study highlights gratitude as a pivotal psychological asset that can enhance an individual’s capacity to positively appraise and cope with career-related stressors, thereby fostering career resilience. This finding enriches the TTSC by demonstrating how positive emotions can function as critical components in the appraisal and coping processes, offering a broader perspective on the theory’s applicability beyond stress and coping to include career development outcomes.

Moreover, the study’s exploration of the moderating effects of personality traits, particularly conscientiousness and extraversion, on the relationship between gratitude and career resilience introduces a significant theoretical advancement. Our study suggests that the impact of gratitude on career resilience and success is not uniform across individuals but is influenced by their inherent personality characteristics. This insight contributes to the personality psychology literature by providing empirical evidence on the role of individual differences in the effectiveness of psychological resources like gratitude. Inferring upon these insights, we also call for a more personalized approach in career counseling and development programs, emphasizing the importance of tailoring interventions to align with individuals’ personality profiles to optimize career outcomes.

Furthermore, the findings offer a compelling argument for the inclusion of positive psychological constructs, such as gratitude, in the discourse on career success and resilience. By evidencing the direct and indirect pathways through which gratitude affects career resilience and success, the study expands the conceptual boundaries of career development theories. Further, it invites future researchers to consider positive psychological resources as integral components of career development models, potentially leading to more holistic approaches that account for the interplay between an individual’s psychological makeup and their professional growth. In doing so, this study paves the way for future empirical investigations and theoretical discussions on the synergistic effects of psychological well-being and career-oriented behaviors in shaping successful career paths.

### Practical implications

This study yields practical implications that extend beyond academic discourse, offering valuable insights for individuals, educational institutions, and career development practitioners. Firstly, these findings suggest that fostering a sense of gratitude among Chinese college students could be instrumental in enhancing their career resilience. We suggest that educators and mentors may consider incorporating gratitude-focused interventions or workshops to promote a positive mindset that facilitates adaptive coping with the challenges inherent in academic and professional pursuits. Additionally, the study advocates for the implementation of career resilience programs tailored to the unique needs of Chinese students. These initiatives could include skill-building workshops, mentorship programs, or counseling services aimed at equipping students with the tools to bounce back from setbacks.

Furthermore, the results emphasize the importance of recognizing the role of conscientiousness and extraversion in shaping career resilience and success. Institutions may consider integrating personality assessments into career development programs to help students identify their strengths and potential areas for growth. This personalized approach can inform targeted interventions to enhance students’ coping strategies and interpersonal skills. The study also recommends that organizations and employers recognize and value these personality traits in the recruitment and professional development processes, acknowledging their potential contributions to career resilience and success.

The study’s implications extend to policy recommendations for educational institutions. Policymakers may consider incorporating positive psychology principles and resilience-building strategies into the curriculum, promoting a holistic approach to student development. This could involve integrating modules on gratitude, resilience, and personality development into existing courses. Moreover, the findings suggest that institutional policies fostering a positive and supportive learning environment could contribute to the overall well-being and success of Chinese college students.

Additionally, career development practitioners are encouraged to tailor their guidance to individual personality traits. Recognizing the moderating influence of conscientiousness and extraversion, counselors and advisors can provide personalized career advice and strategies that align with students’ unique strengths and characteristics. Mentorship programs could be designed to match students with mentors who share similar personality traits, fostering a more effective and tailored support system.

In conclusion, this study’s practical implications underscore the potential for targeted interventions and policy changes that prioritize gratitude cultivation, resilience-building, and the recognition of personality traits in the career development journey of Chinese college students. These recommendations aim to create a more supportive and adaptive educational environment, ultimately contributing to the holistic success and well-being of students as they embark on their professional paths.

### Limitations and future directions

While this study provides valuable insights into the relationships between gratitude, personality traits, career resilience, and success among Chinese college students, several limitations should be acknowledged. Firstly, the cross-sectional nature of the data prevents the establishment of causality. Future research employing longitudinal designs could provide a more nuanced understanding of the dynamic interplay between these variables over time. Secondly, the study relies on self-report measures, which may introduce common method bias and social desirability effects. Incorporating objective measures or alternative assessment methods could enhance the robustness of the findings. Additionally, the sample predominantly consists of Chinese college students, limiting the generalizability of the results. Future research could explore these relationships across diverse cultural and educational contexts to enhance the external validity of the findings. The study also focuses on a specific set of personality traits (conscientiousness and extraversion). While these traits were chosen based on theoretical relevance, future research could investigate the influence of other personality traits to provide a more comprehensive understanding of the role of individual differences in career development (Obrenovic et al., 2022). In addition, future studies should explore the impact of AI-based learning on enhancing gratitude and career success among students (Lăzăroiu, 2017; Peters et al., 2023). Additionally, investigating occupational flexibility as a factor in career resilience offers a promising direction for understanding dynamic career paths. Lastly, the study does not delve into the potential influence of external factors such as socio-economic background or cultural influences, which may play a role in shaping career outcomes. Considering these factors in future research could contribute to a more nuanced understanding of the complexities influencing career development among college students.

## Data availability statement

The raw data supporting the conclusions of this article will be made available by the authors, without undue reservation.

## Ethics statement

The studies involving humans were approved by Quality Enhancement Cell (QEC), Nanning College of Technology, China. The studies were conducted in accordance with the local legislation and institutional requirements. The participants provided their written informed consent to participate in this study.

## Author contributions

KZ: Conceptualization, Data curation, Formal analysis, Investigation, Writing – original draft, Writing – review & editing. WJ: Formal analysis, Methodology, Validation, Visualization, Writing – original draft, Writing – review & editing.
